# Short-acting β2-agonists (SABA) bronchodilator sales and outdoor mould in central France

**DOI:** 10.1186/s13601-019-0296-1

**Published:** 2019-10-25

**Authors:** D. M. Caillaud, S. Martin, C. Ségala, N. Dupuy, M. Thibaudon, D. Muti

**Affiliations:** 1 Service de Pneumologie-Allergologie, Hôpital Gabriel Montpied, Clermont Auvergne University, Montalembert Street, 63003 Clermont-Ferrand, France; 2 SEPIA-Santé, 56150 Baud, France; 3 Réseau National de Surveillance Aérobiologique, 69690 Brussieu, France

**Keywords:** *Alternaria*, *Aspergillus–Penicillium*, Asthma, Mould, Outdoor, Spores, Pharmacoepidemiology

## Abstract

**Background:**

Temporal ecological studies have shown that outdoor moulds are associated with severe asthma exacerbations, as emergency department visits or hospitalisations. The aim of this was to assess the associations between daily sales of short-acting β2-agonists (SABA), a specific and frequent treatment for control of mild asthma exacerbations in children and young adults, and outdoor mould concentrations in the central France area over a 5 year period.

**Methods:**

The relationship between daily changes in mould (25 species) concentrations and daily SABA sales within a population of patients aged 6 to 39 years in a middle-size town of central France (approximately 127,000) was obtained from social security database and analysed with generalized additive models, taking into account confounding factors (air pollution, weather conditions, pollen counts and trend).

**Results:**

Daily SABA sales (mean, SD) rose from 17.3 (9.7) in 2010 to 22.7 (12) in 2015. The relative risk (RR [95% CI]) of SABA sales associated with an interquartile increase in mould concentration was significant in the whole population for *Alternaria* 1.06 [1.002–1.12]. When the influence of age and sex was accounted for, the relationship was significant only in 6–12 years old males for *Alternaria* 1.21 [1.04–1.41] and *Aspergillus*–*Penicillium* 1.08 [1.04–1.12].

**Conclusions:**

Daily SABA sales are positively associated with *Alternaria* spores in the general population of children and young adults. The association between daily SABA sales and temporal changes to *Alternaria* and *Aspergillus*–*Penicillium* in male children indicate that outdoor moulds contribute to asthma morbidity.

## Introduction

Asthma exacerbations, as shown by emergency department visits or hospitalizations, are associated with outdoor moulds, but it is difficult to identify which mould taxa are responsible for these severe and infrequent events. In contrast, short-acting β2-agonists agonists (SABA) are widely used by patients experiencing mild asthma exacerbations, which translates into increased sales. SABA sales have been successfully used to illustrate the relationship between asthma and outdoor air pollution [1]. This study will therefore test, over a 5 year period, the relationship between exposure to individual outdoor mould taxa in the general population of central France, differentiated by age and gender, with SABA sales.

## Methods

Records of all SABA treatments prescribed for people living in the demographically stable Clermont-Ferrand area (approximately 285,000 inhabitants) were provided for the study period (2010–2012; 2014–2015) from the public health insurance database, which covers 80% of the French urban population. Owing to missing data due to technical reasons, 2013 was omitted. The health outcome was defined as the number of “cases” per day, whereby a case is defined as the reimbursement of a SABA treatment. SABA sales were excluded for children < 6 years because of the uncertainty of asthma diagnosis [2] and for adults > 39 years, because they are also used for acute exacerbations of COPD. This study was entirely anonymous and so approval from the French Ethics Committee was not required. Mould samples were collected daily from mid-February to early October, as described previously [3]. Molds and pollens were sampled on the rooftop of a building of the University Hospital, with no nearby buildings obstructing air circulation. Samples for microscopic analysis were collected with a coat paper using a 7 days recording volumetric trap of the Hirst design, at a suction rate of 10 L/min. The tape representing 1 week of sampling was cut into 7 fragments, each representing 1 day, and transferred to a glass slide. After application of a reactive colouring solution, the mean spore concentration, expressed as total number of spores per cubic meter of air (spores/m^3^), was determined using 400× optical microscopy along 1 longitudinal sweep per slide. Spores were identified at the genus level only, since in most cases it was not possible to identify spore species. *Cladosporium* and *Alternaria* taxa collected for 5 years, other mould taxa for 3 years. Data on influenza epidemics was obtained from the national network for transmissible disease surveillance. Air pollution (PM_10_, O^3^, NO_2_), meteorological data, age and sex were available from the database.

Data were analyzed by overdispersed Poisson regression with generalized additive models (GAMs). Using non-parametric smoothing functions, GAMs allow flexible control of the effect of trend and seasonal components and of confounding factors whose relationship with asthma is not linear. The construction of the model began with the introduction of the long-term trend and seasonal variations, using a cubic smoothing spline of the day of the study. Holidays, days of the week and influenza occurrence were then introduced as dummy variables. Quantitative meteorological, pollution and pollen variables were introduced as penalized cubic splines, with different lags tested. Finally, mould counts were introduced in the form of penalized cubic splines, with lags of up to 7 days. The effect of mould on asthma in the short term is expressed as a relative risk (RR and 95% CI) for an increase of the interquartile range of grains (statistical program: SM).

## Results

**Fig. 1:**
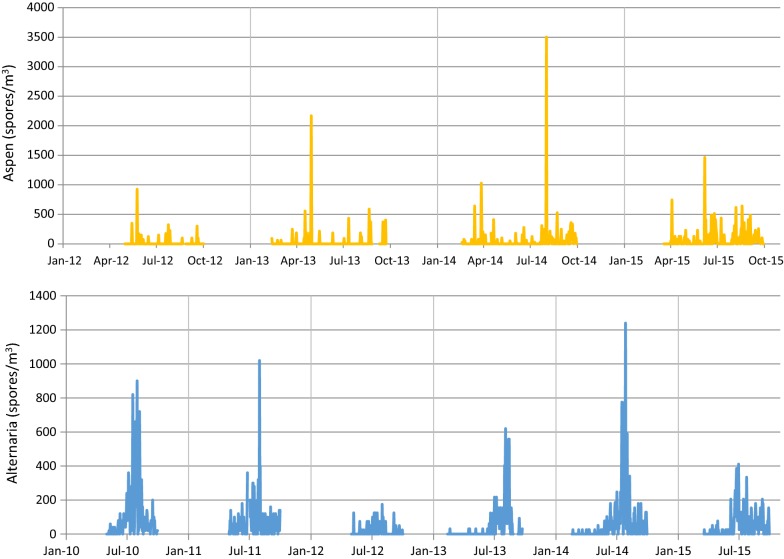
*Aspergillus–Penicillium* and *Alternaria* sporulation from 2010 to 2015

**Fig. 2:**
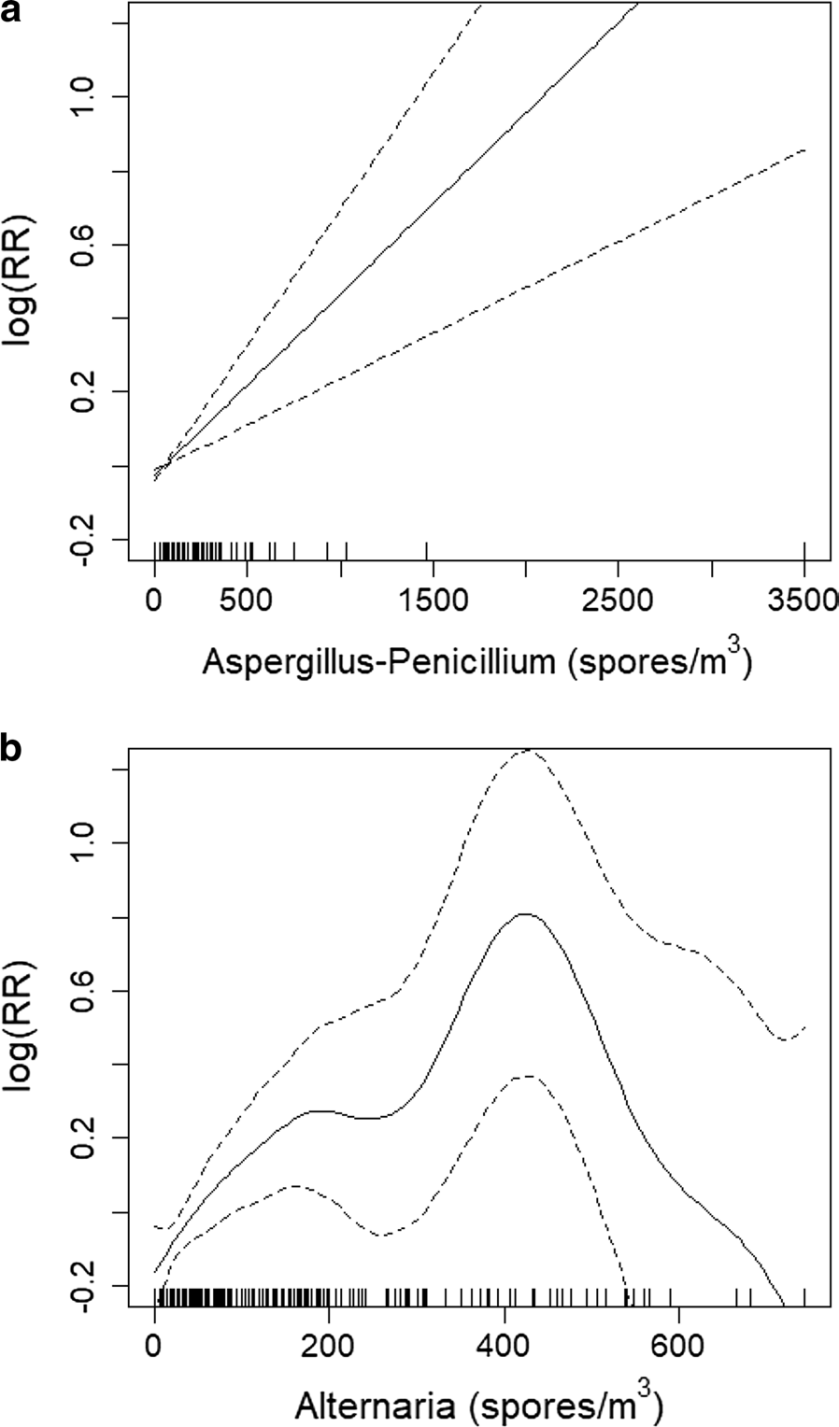
**a** *Aspergillus–Penicillium* and SABA sales in 6–12 years old male children.** b** Alternaria and SABA sales in 6–12 years old male children. Shape of the mold-prescribed SABA sales curve from the GAMM models for *Aspergillus–Penicillium* and *Alternaria* in 6–12 years old male children. The mold-prescribed allergy SABA sales relationship (expressed as natural logarithm of the relative risk ratio [Log (RR)] can be read as the prescribed SABA sales increase on the y-axis for a mold increase on the x-axis

Table 1 shows the 10 moulds present in outdoor air for more than 90 days (among 25 individual taxa identified) and the trends in the daily concentrations of *Alternaria* and *Aspergillus–Penicillium* are represented in Fig. 1. The daily SABA sales (mean, SD) for 6 to 39 year old subjects rose significantly from 17.3 (9.7) in 2010 to 22.7 (12.3) in 2015. Across the entire population, the RR [95% CI] of SABA medication sales associated with an interquartile increase in spore concentration was significant for *Alternaria* 1.06 [1.002–1.12]. When the influence of age and sex was considered (Table 2), the relationship was significant only in male children aged 6–12 years for *Alternaria* and *Aspergillus*–*Penicillium*. The shapes (with 95% CI) of the mould-SABA sale relationship in male children aged 6–12 for *Aspergillus–Penicillium* (Fig. 2a) and *Alternaria* (Fig. 2b) suggest that the effect of *Aspergillus–Penicillium* on SABA sales is linear whereas the effect of *Alternaria* spores is approximately linear up to a saturation point of approximately 400 spores/m^3^.”

**Table 1:** Outdoor mould taxa in Clermont-Ferrand, 2010–2012 and 2014–2015

Fungal spore taxa	Number of days^a^	Number of spores/m^3^					
		Mean (SD)	Min	Q1	Median	Q3	Max
*Alternaria*	507	108 (139)	20	26	60	124	1240
*Aspergillus–Penicillium*	171	183 (320)	26	51	103	206	3503
*Cladosporium*	854	3397 (4417)	20	771	2048	4000	36,263
*Epicoccum*	248	69 (61)	25	26	51	77	437
*Erysiphe*	195	54 (45)	25	26	31	75	283
*Ganoderma*	265	109 (75)	26	51	103	154	488
Helicomyces	91	68 (108)	25	26	31	62	675
*Myxomycetes*	176	108 (173)	26	26	51	103	1388
*Torula*	164	46 (31)	25	26	26	51	231
*Ustilago*	189	170 (252)	26	31	77	186	1645

^a^Number of days with detectable levels of each mold across all monitored days

**Table 2:** Outdoor moulds and SABA sales by age and gender, 2010–2012, 2014–2015

Mould	Lag (days)	IQR	Sex	Age (years)			
				6–12		13–39	
				RR	CI 95%	RR	CI 95%
*Alternaria*	0–2	66	*M*	*1.21*	*[1.04–1.41]*	1.001	[0.993–1.008]
			F	1.04	[0.99–1.1]	1.05	[0.97–1.13]
*Aspergillus–Penicillium*	1	154	*M*	*1.08*	*[1.04–1.12]*	1	[0.999–1.00]
			F	1	[0.999–1.001]	0.98	[0.93–1.03]

Significant results (p < 0.05) are in italic characters. Lag: associations were estimated for optimal lag defined according to the associations reported for individual lags. For example: *Alternaria* Lag 0–2 corresponds to the mean *Alternaria* concentration for D0, D1 and D2

## Discussion

Our study, using SABA sales as a very specific marker for asthma attacks within the 6 to 39 year-old population (mean of 20 SABA sales per day across several years), reveals an association between *Alternaria* spore concentration and daily SABA sales across the general population. Epidemiological studies examining the relationship between outdoor *Alternaria* and asthma in people not known to be sensitized to *Alternaria,* are scarce. A Californian study [4] demonstrated an association between asthma symptom scores and *Alternaria* levels, while an Australian study found *Alternaria* exposure to be significantly associated with asthma hospitalization [5]. In Saint-John, Canada (asthma ED visits of 3.5 per day), the concentration of *Alternaria* spores was associated with a 4.5% increase in ED visits [6]. Our study, by showing that outdoor *Alternaria* moulds are associated with a 6% increase in SABA sales, confirms the latter result on a larger sample of the general population.

In addition to *Alternaria*, the association between outdoor individual spores and SABA sales was significant for *Aspergillus*–*Penicillium* spores in children. Only 1 Canadian study has reported the effects of outdoor *Aspergillus*–*Penicillium* spores on asthma. In Ontario, an adjusted 2.34% significant increase in child ED visits was observed with an increase in *Aspergillus*–*Penicillium* spore concentration [7].

Among children, the relationship between SABA sales and mould concentrations was observed only in boys. Only 1 study reported a significant increase in daily hospitalizations in boys in association with two outdoor fungal taxa, deuteromycetes (which include *Alternaria* and *Aspergillus*–*Penicillium*) and basidiomycetes [8].

The main strength of the present study lies in the extremely large regionally representative dataset comprising subjects between 6 and 39 years of age, and living within 15 km of the spore trap. The concentrations of outdoor spores, with *Cladosporium* predominating, are in line with other studies performed in European temperate climates. We used appropriate statistical tools to reveal the shape of the curve for every mould spore studied, while adjusting for potentially confounding factors (such as air pollution, meteorology and pollens). The lack of knowledge about mould sensitization in our general urban population is a potential limitation. As the genera *Aspergillus* and *Penicillium* are morphologically indistinguishable, laboratories list them together as *Aspergillus*–*Penicillium* type. Outdoor mould effect will vary by climatic region [9]. Data on rhinovirus and respiratory syncitial virus, which are known, confounders of asthma exacerbations were unfortunately not available in France during the study period.

In conclusion, this study, performed in central France over a 5-year period, showed that daily changes of *Alternaria* outdoor spores were associated with increased SABA sales in the general population of children and adults up to the age of 40. Outdoor *Aspergillus*–*Penicillium*, and *Alternaria* mould spore concentrations were associated with increased SABA sales in boys aged 6 to 12 years. These results suggest that moulds contribute to mild asthma exacerbations in the general population. Further studies in other geographical areas with different climatic conditions are needed to complement these results.

## Data Availability

Contact C Segala for an anonymized database of this study.
